# Evaluation of neighborhood deprivation and store characteristics in relation to tobacco retail outlet sales violations

**DOI:** 10.1371/journal.pone.0254443

**Published:** 2021-07-16

**Authors:** David C. Wheeler, Elizabeth K. Do, Rashelle B. Hayes, Colleen Hughes, Bernard F. Fuemmeler

**Affiliations:** 1 Department of Biostatistics, Virginia Commonwealth University, Richmond, VA, United States of America; 2 Department of Health Behavior & Policy, Virginia Commonwealth University, Richmond, VA, United States of America; 3 Department of Psychiatry, Virginia Commonwealth University, Richmond, VA, United States of America; 4 Virginia Department of Behavioral Health and Developmental Services, Richmond, VA, United States of America; 5 Massey Cancer Center, Virginia Commonwealth University, Richmond, VA, United States of America; University of Calfornia San Francisco, UNITED STATES

## Abstract

**Introduction:**

Regulations of the sale of tobacco products to minors have been effective at reducing adolescent tobacco use overall. However, these efforts may not be uniformly enforced in all areas, creating uneven protection against adolescent smoking. Knowledge regarding factors associated with tobacco retail outlet (TRO) violations could help inform better enforcement strategies.

**Methods:**

In this study, we used Bayesian index regression models to determine if tobacco sales to minors violations across Virginia (2012–2021) were related to store characteristics and neighborhood deprivation and identify geographic areas at significantly elevated risk for violations after adjusting for these factors.

**Results:**

Results show that there were multiple factors associated with a higher likelihood of tobacco sales violations. Store type was an important factor, as grocery stores and pharmacies had significantly lowered likelihood of violations compared with convenience stores. Being located near another TRO was significantly associated with increased risk of sales to a minor. Neighborhood deprivation was also positively associated with TRO sales violations. Further, there were statistically higher likelihood of sales violations occurring in specific areas (e.g., southwest and southeast) of the state that were not explained by neighborhood deprivation and store attributes.

**Conclusions:**

Together, results highlight the need to better understand where and why TRO sales violations are occurring in order to improve efforts aimed at monitoring and remediating TRO sales violations.

## Introduction

Tobacco use is a cause of chronic diseases and cancer [1, 2]. Initiation begins during adolescence [3–5] and most teens who use tobacco will continue to use into adulthood. Thus, halting initiation of adolescent tobacco use is of high public health importance [6].

Imposing age restrictions for the sale of tobacco products is critical to reducing adolescent tobacco use [6, 7]. The Synar Inspection Program and the Food and Drug Administration (FDA) Tobacco Retail Compliance Program have been keystone programs in this effort. Both of these programs support efforts to assess compliance with laws and regulations prohibiting the sale to minors, primarily through use of random inspection checks [8–10]. In general, these programs have resulted in significant reductions in the prevalence of smoking among minors [11, 12]. However, what these data also show is that there may be significant variation in compliance with laws restricting sales to minors across states, communities, and by store types [9, 12]. Uneven compliance with tobacco sales to minors has the potential to create geographic disparities in smoking rates. Determining to what degree store-type and area-level factors are associated with violations could help inform public health efforts to more evenly reduce illicit tobacco sales to minors in all regions, thereby reducing geographic disparities in youth smoking rates.

Several studies have examined area-level characteristics and retail violations for sales to minors. One of these studies found significant positive associations between rates of retail violations for sale to minors and several census tract variables including percent African-American population, percent Hispanic population, and percent of persons living in poverty using FDA compliance check data across the United States [13]. A similar study of sales to minors by different tobacco products at the ZIP Code level across the United States found that percent living in poverty was significantly and positively associated with smokeless tobacco and cigar sales violations, while percent African-American population was significantly and positively associated with cigar and cigarette sales violations [14]. In a study in Florida, block groups with youth sales violations were significantly more likely to be associated with lower per capita income, higher percent Hispanic population, and lower percent African-American population compared with block groups with no violations [15]. A study of rates of sales to minors at the ZIP Code level in Los Angeles, California found that lower income areas were more likely to have higher rates of underage tobacco sales [16]. Finally, a study of tobacco store sales to minors and neighborhood characteristics at the census tract level across the United States found that the percent African-American population and percent Latino population were significantly and positively associated with the likelihood of sales to minors [17]. While these studies revealed associations with neighborhood characteristics and sales to minors, there were some limitations. None of the studies included store-level attributes in their analyses, most [13–16] conducted their analysis at the area level (e.g., ZIP Code, block group, census tract) and as such were ecological analyses, and most [13–17] did not attempt to identify statistically elevated areas at risk for sales violations. Ecological studies may overestimate or misattribute the area-level factors for sales violations that are actually occurring related to individual-store level factors. Also, it is critical to identify areas elevated in risk, as this information could help advance precision population health interventions: for example, increasing resources to help mitigate sales violations through store-owner training in high-risk areas.

To date, only one study has examined store-type and area-level factors associated with individual-level youth sales violations. In that study, conducted within Washington, District of Columbia, investigators found that illicit tobacco sales to minors were more common at gas stations, at outlets displaying exterior tobacco advertisements, and at outlets located closer to high schools located in communities with a higher majority African-American population living within those communities [9]. While this study provided novel information about the potential factors related to violations, it was limited by a focus on one urban area and did not identify geographic areas (e.g., census tracts, ZIP Codes) of elevated risk.

Extending this line of research, we analyzed the likelihood of tobacco sales violations across a larger geographic region (the state of Virginia) with a mix of rural and urban localities using Bayesian index regression models. We aimed to determine the store attributes and area-level characteristics associated with tobacco sales to minors violations, and identify geographic areas with elevated risk of sales violations after adjusting for these factors. We hypothesized that there would be geographic spatial structure in the likelihood of violations, that such structure could be linked with area-level characteristics such as neighborhood deprivation, and that sales violations would vary by store type. In this paper, deprivation means deprived of resources, capital, and opportunities over time, which could be due to systematic racism and discriminatory practices. In the United States, concentrated racial segregation has resulted from a history of discrimination and racist housing and urban development policies that have led to the devaluation and deprivation of resources within neighborhoods.

## Materials and methods

### Data sources

We obtained data from a variety of sources, including: 1) the Virginia Counter Tools database that includes information on tobacco retail outlets (TROs), Synar inspection data, and the Food and Drug Administration Tobacco Retail Compliance Check Program, 2) the American Community Survey (ACS), and 3) Behavioral Risk Factor Surveillance System (BRFSS). Using the Counter Tools program, the Virginia Department of Behavioral Health and Developmental Services created an accurate state-wide tobacco retailer list in 2005, which has been updated biannually [18]. Downloadable data on retailers are available through Counter Tools Store Mapper and Audit Center [19] and includes information on: retailer locations and types, enforcement, and Synar compliance assessments from 2013 to 2021. FDA compliance checks, conducted independently from Synar by the Virginia Department of Alcohol and Beverage Control (ABC), were obtained from the FDA website for years 2012 through 2021 [10]. For both the Synar and FDA programs, underage buyers (aged 16–17 years) make controlled purchases of tobacco products under the direction of law enforcement officers at randomly selected TROs. The ACS is administered annually by the US Census Bureau to three million households. Estimates are currently available for one-year or five-year periods. BRFSS is administered by the Centers for Disease Control and Prevention and is a telephone survey that collects data about health-related risk behaviors and chronic health conditions for more than 400,000 adults each year in the United States.

### Measures

#### Violations

Both Synar inspections and FDA Tobacco Retail Compliance Checks determine compliance with laws and regulations that prohibit the sale of tobacco products to any person under the legal age of sale using underage buyers (age 18 years until 2019 when the federal legal age of sale was increased to 21 years). The FDA compliance checks also require retailers to examine photographic identification of anyone under age 27 who attempts to purchase these products, and bans vending machines and self-service displays, except in facilities where the retailer ensures that no person younger than the legal age of sale is present or permitted to enter at any time. Visited retailers are reported as either having had any violation (e.g. noncompliant) or no violation. The FDA program records the type of violation, while Synar only records sales to minors as a violation. For this analysis, we analyzed only sales to minors as the violation type, which is the overwhelming majority (96%) of violations recorded by the FDA. The federal law increasing tobacco legal age of sale to 21 years allowed for a mandatory 3-year phase-in implementation period, such that violations were still counted for sales to minors under the age of 18 years in 2019 and 2020. Regarding the sampling methodology of inspections, both FDA and Synar inspections in Virginia utilize a list frame sampling methodological approach. While FDA inspections contracts do not require a statistically representative sample (e.g., probability-based) of tobacco outlets, FDA inspectors are encouraged to conduct inspections in a variety of different locations, outlet types, and communities, including minority communities under Section 105 of the Tobacco Control Act [20]. The sample frame coverage target is 90%, but the number of enforcement visits varies by year depending upon available funding and resources to conduct inspections. FDA funding contracts to support enforcement visits in Virginia was significantly reduced in 2018 [21].

#### Socioeconomic Status (SES) variables

We considered five-year (2012–2016) ACS estimates of 14 variables at the census tract level as candidates for estimating neighborhood deprivation with Bayesian index models. The variables were: Gini index of income inequality, percent Black population, percent without a bachelor’s degree, percent of families in poverty, percent of households with public assistance, percent vacant housing units, percent renter occupied housing units, median household income, per capita income, median gross rent, median monthly housing costs, percent of housing units with a mortgage, percent Hispanic population, and percent US citizen. We selected these candidate variables based on our experience estimating a neighborhood deprivation index (NDI) for TRO density using a Bayesian index model [13–17, 22], as well as findings from previous studies that have examined neighborhood characteristics and sales violations [9, 13–17]. We included percent Black population in the index because it is a measure of Black racial segregation resulting from a history of discriminatory practices that have led to the devaluation and deprivation of resources within segregated neighborhoods.

#### Store type

TROs were categorized into 10 store types: convenience store with or without gas; drug store or pharmacy; beer, wine, or liquor store; grocery store; mass merchandiser; tobacco shop; hookah lounge; e-cigarette/vape shop; bar or restaurant; and other store type not listed. For regression models that estimate store type effects, we combined beer, wine, or liquor store; hookah lounge; and bar or restaurant into the other store type due to small numbers of these categories.

#### Store attributes

The proximity of the TRO to a school was recorded in Counter Tools as “yes” if the store was within 1,000 feet of a school and “no” if otherwise. Having another TRO located within 500 feet of the TRO was also recorded as a binary variable. Data on TROs accepting benefits from the Special Supplemental Nutrition Program for Women, Infants, and Children (WIC) or the Supplemental Nutrition Assistance Program (SNAP) were also obtained from Counter Tools [19] based on the Virginia Department of Health’s Authorized Retailer Listing of WIC retailers [23] and the US Department of Agriculture Food and Nutrition Service’s SNAP Retailer Locator [24].

#### Percent current smokers

We used estimates from the Virginia Department of Health on the percent of current smokers per county in Virginia based on the BRFSS 2013–2017 annual survey data. The current smoker variable was calculated for individuals responding to the BRFSS annual survey as “yes” for those who now smoke every day or who now smoke some days. We assigned the appropriate values to TROs based on the county that the TRO was located within. We scaled the percent current smokers variable so that a one-unit increase represented a one standard deviation increase.

#### TRO density

We calculated the density of TROs visited for compliance checks by census tracts in the time period 2012–2021 by dividing the count of TROs by the number of households in the census tract. We scaled the TRO density variable so that a one-unit increase represented a one standard deviation increase.

### Statistical analysis

#### Bayesian index models

To model the likelihood of a sales violation among the TROs that had been visited for compliance checks, we used Bayesian index regression models [22, 25, 26]. This type of model allowed us to estimate the NDI while including store-level attributes and area-level random effects for unexplained risk at the census tract level. Assuming that the outcome variable of the number of sales violations at a TRO was *y*_*i*_~*Binomial*(*p*_*i*_, *n*_*i*_) with probability *p*_*i*_ and number of visits *n*_*i*_, the basic form of the Bayesian index model is ${logit(p}_{i})=\beta_{0}+\beta_{1}\left(\sum_{j=1}^{C}w_{j}q_{ij}\right)+\alpha z_{i},$ where *β*_0_ is the intercept, *β*_1_ is the effect for the neighborhood deprivation index, and *α* is a vector of regression coefficients for store characteristics in the vector *Z*_*i*_. The neighborhood deprivation index is a weighted combination $\sum_{j=1}^{C}w_{j}q_{j}$ of the quantiles *q*_1_,…,*q*_*c*_ of the SES variables *x*_1_,…,*x*_*c*_, where the weights *w*_1_,…,*w*_*c*_ were estimated in the model. The weight *w*_*j*_ represents the relative importance of the *j*_*th*_ SES variable in the index. Quantiles (deciles) of the SES variables are used to account for different scales of the variables, de-correlate the variables, limit the effect of outliers, and acknowledge uncertainty in the ACS covariates.

The Bayesian index model specification is complete with the definition of prior distributions for the priors. The index weights are given a Dirichlet prior with parameters *α* = (*α*_1_,…,*α*_*C*_). The Dirichlet prior is used because it assures that the SES variable weights *w*_*j*_∈(0,1) and $\sum_{j=1}^{C}w_{j}=1$. The intercept follows an improper uniform distribution *α*~*dflat*(), which means that it is a uniform prior distribution that extends over the whole real line. The index regression coefficient has a vague normal prior *β*_1_~*Normal*(1, *τ*_1_) with precision $\tau_{1}=1/\sigma_{1}^{2}$ and *σ*_1_~*Uniform*(0,100). Covariate regression coefficients in the *α* vector receive a vague normal prior of *α*_*k*_~*Normal*(1, *τ*_*k*_) with precision $\tau_{k}=1/\sigma_{k}^{2}$ and *σ*_*k*_~*Uniform*(0,100). These are non-informative priors that contain minimal information and allow the data to inform about the joint posterior distribution of the model parameters. We conducted the model building in steps and describe the model building process in the following sections.

#### Neighborhood Deprivation Index (NDI)

We estimated a crude model with only the NDI (model 1) considering the candidate SES variables from the ACS at the census tract level. To do so, we conducted univariate correlation analysis of the SES variables with the probability of a sales violation to determine the direction of association of each variable with the outcome. We then inverted the variables with a negative association using the transformation max(x)–x to have all variables in a positive direction of association with the outcome. We next fitted a series of crude models of the NDI starting with deciles of all the variables in the index and then systematically decreasing the number of variables by removing variables with small weight. By comparing the deviance information criterion (DIC) among the candidate models, we found that the best fitting model had a parsimonious index with the following five variables: percent Black population, percent of families in poverty, Gini index of income inequality, percent without a bachelor’s degree, and inverse percent Hispanic population (i.e., non-Hispanic population).

#### Store attributes

To estimate the effects for store and area characteristics, we adjusted the NDI model for store type, accepting SNAP, accepting WIC, being located near a school, being located near another TRO, percent of current smokers per county, and TRO density (model 2). Convenience store with or without gas was the reference category for store type, and there was also a category for TROs missing store type.

#### Census tract effects

To model unexplained risk of a sales violation at the area level and identify areas with increased likelihood of a sales violation, we added to the adjusted model exchangeable random effects for census tracts (model 3) and spatial random effects for census tracts (model 4). The form of this model is ${logit(p}_{i})=\beta_{0}+\beta_{1}\left(\sum_{j=1}^{C}w_{j}q_{ij}\right)+\alpha z_{i}+v_{k},$ where *v*_*k*_ is the random effect for the *j*th census tract that the *i*th TRO is located within. Both of these models (models 3 and 4) include terms for sales violation risk at the census tract level, but model 4 allows the risk to be correlated over space. In other words, sales violation risk in one census tract is assumed to be related to that of the adjacent census tracts. This correlation over space is specified through an intrinsic conditional autoregressive (CAR) prior, *v*_*k*_~*CAR*(*τ*_*v*_) with precision *τ*_*v*_. The exchangeable random effects have a prior of *v*_*k*_~*Normal*(0, *τ*_*v*_).

#### Model fitting and evaluation

We used Markov chain Monte Carlo (MCMC) to estimate the model parameters with a total of 40,000 iterations from one chain and a thinning parameter of one, where the first 20,000 iterations were used for burn-in. We checked for convergence of the MCMC algorithm for parameters of interest using the Geweke convergence diagnostic [27]. A parameter was considered to have converged if its diagnostic absolute value was less than 2. The 95% credible interval was used to determine the statistical significance of the regression effects, with there being a significant effect if the interval did not contain the null odds ratio (OR) value of 1. We fit the Bayesian models using WinBUGS 1.4.1 [28] with the R2WINBUGS package in the R computing environment. We compared the goodness of fit of the models using the DIC and the complexity with the effective number of parameters *pD*. To identify geographic areas associated with increased likelihood of a sales violation, we calculated exceedance probabilities of odds ratio for census tracts exceeding the null values of 1.0. Those census tracts with an exceedance probability > 0.80 were considered most associated with increased likelihood of a sales violation.

## Results

**Fig 1:**
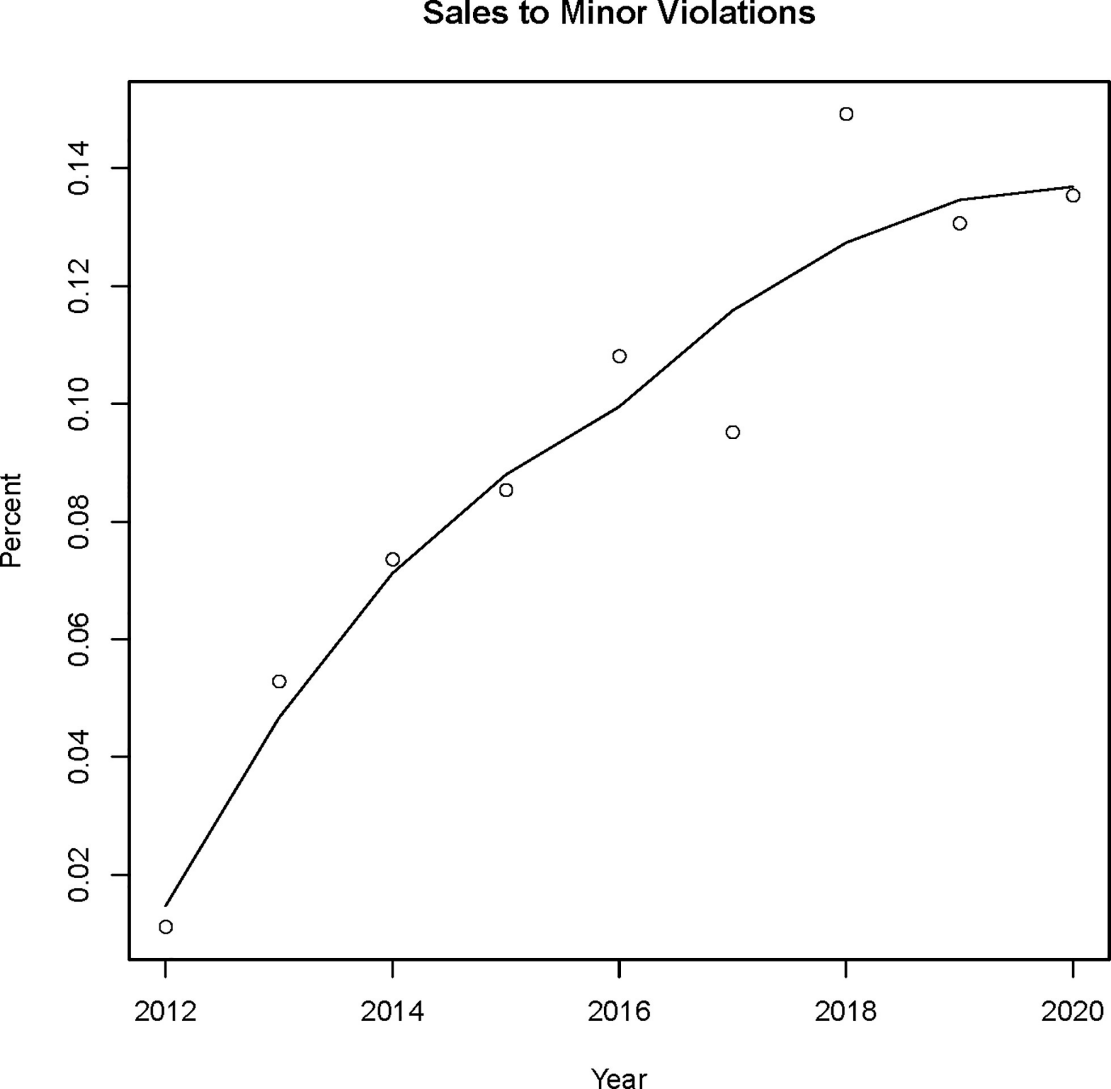
Percent of compliance check visits with a violation for a sale to a minor by year in 2012–2020 with a loess best fit curve.

**Fig 2:**
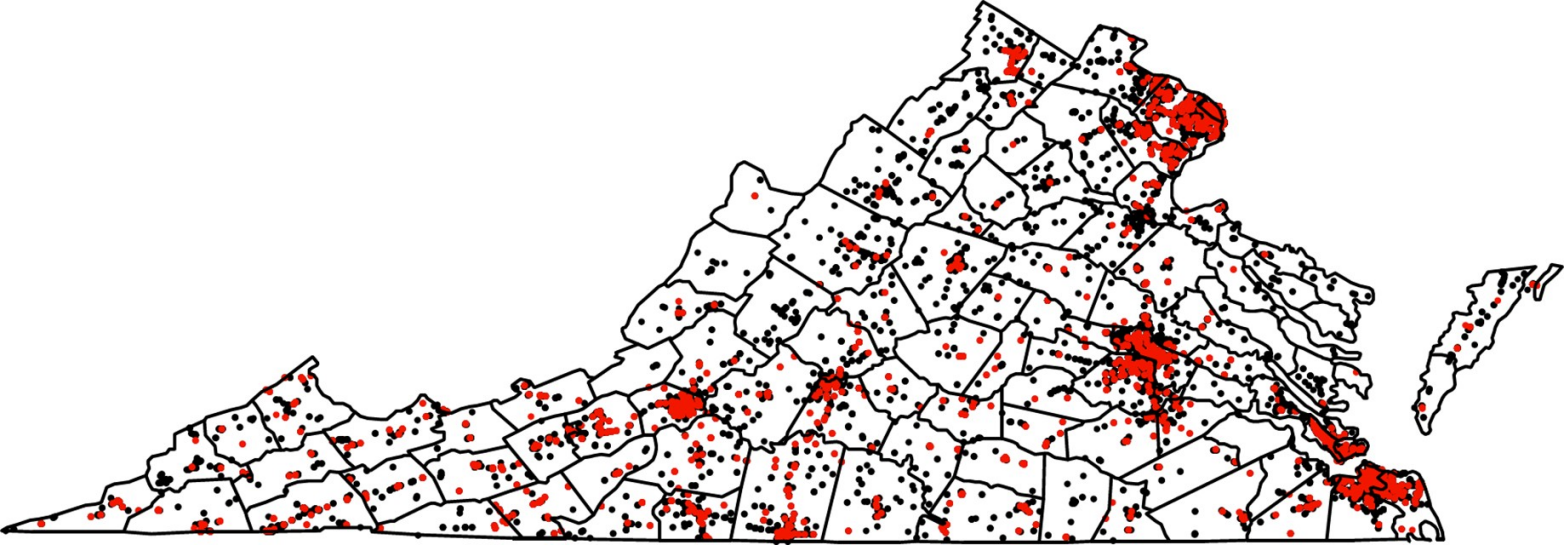
Locations of compliance checks (black) and sales to minor violations (red) shown with county boundaries in Virginia.

There were 15,766 compliance check visits to TROs in the time period 2012–2021. Out of these visits, there were 1,535 violations for sales to a minor, for an overall violation percent of 9.74%. There were 7,166 TROs with compliance checks in the period 2012–2021. After matching to the ACS and BRFSS data, there were 7,105 TROs with complete area data to be used for modeling the probability of a sales violation. The counts of compliance check visits, sales to minor violations, and percent of visits with a sales violation by year are listed in Table 1. The years 2016 and 2018–2020 were above the overall average in percent violations. There was a notable trend in the violation percent over time, excluding 2021 due to being an incomplete sampling year (Fig 1). The loess fit line shows a generally increasing trend in the percent of visits leading to a sales violation. The locations of the sales violations to minors (red) compared with the compliance checks (black) generally show more violations in more populated areas such as Northern Virginia, Richmond, and Virginia Beach, however, southwestern Virginia also appears to have a concentration of violations (Fig 2).

**Table 1:** Counts of compliance check visits, sales to minor violations, and percent of visits with a sales violation by year and overall in 2012–2021.

Year	Visits	Violations	Percent
2012	90	1	1.11%
2013	1553	82	5.28%
2014	1523	112	7.35%
2015	2801	239	8.53%
2016	3295	356	10.80%
2017	3521	335	9.51%
2018	2004	299	14.92%
2019	712	93	13.06%
2020	133	18	13.53%
2021	134	0	0.00%
Total	15766	1535	9.74%

The percent of compliance checks with a sales violation varied by store type, with convenience stores, tobacco shops, vape shops, and other stores having higher than average violation rates (Table 2). Other stores and tobacco shops had the highest violation rates, but convenience stores accounted for the most visits and most violations. Compliance checks with a sales violation were more likely to be located within 500 feet of another tobacco retailer than checks without a violation (79.9% vs. 76.7%) and were more likely to be located within 100 feet of a school than checks without a violation (13.4% vs. 11.7%).

**Table 2:** Counts of compliance check visits, sales to minor violations, and percent of visits with a sales violation by store type in 2012–2021.

Store Type	Visits	Violations	Percent
Convenience Store	8544	883	10.33%
Grocery	1747	153	8.76%
Pharmacy	921	51	5.54%
Mass Merchandiser	816	67	8.21%
Tobacco Shop	371	42	11.32%
Vape Shop	161	17	10.56%
Bar or Restaurant	59	5	8.47%
Beer, Wine, Liquor store	20	1	5.00%
Hookah Lounge	10	1	10.00%
Other Store	173	26	15.03%
Missing	2944	289	9.82%
Total	15766	1535	9.74%

The estimated odds ratios (ORs) and 95% credible intervals (CIs) for the Bayesian index regression models reveal several interesting findings (Table 3). Regarding neighborhood deprivation, the index had a significant positive association with sales violations with an OR = 1.03 on its own (model 1). This OR is for a one-unit increase in the NDI and because deciles of the variables were used, a 4-unit increase in the index (e.g., first to fifth decile of NDI) is associated with an OR = 1.12 or 12% increase in risk in a sales violation and a 9-unit increase in the index is associated with a 30% increase in risk of a sales violation. In the adjusted model (model 2) and adjusted model with independent random effects (model 3), the index OR = 1.04 and was significant according to the 95% credible interval. Only in the adjusted model with spatial random effects (model 2) was the NDI not significant. The model goodness-of-fit according to the DIC improved from the crude model when adding other store characteristics and also when adding census tract random effects, even with the greater model complexity as indicated by the *pD* (Table 3). The spatially correlated random effects led to a more substantial improvement in model fit than did the exchangeable random effects, and in fact the model with spatial random effects had the best fit overall.

**Table 3:** Odds ratio and 95% confidence intervals from Bayesian index regression models for the probability of a TRO sales violation.

	Models			
Term	Crude	Adjusted	Independent Random Effects	Spatial Random Effects
Deprivation Index	**1.03**	**1.04**	**1.04**	1.01
	1.01, 1.06	1.00, 1.08	1.00, 1.08	0.98, 1.06
Pharmacy		**0.51**	**0.51**	**0.50**
		0.38, 0.69	0.37, 0.68	0.37, 0.67
Grocery		**0.81**	**0.81**	**0.80**
		0.66, 0.99	0.65, 0.99	0.65, 0.99
Mass Merchandiser		0.79	0.79	0.80
		0.60, 1.01	0.60, 1.02	0.60, 1.03
Tobacco Shop		1.07	1.08	1.10
		0.78, 1.47	0.79, 1.49	0.80, 1.52
Vape Shop		1.00	1.04	1.09
		0.61, 1.56	0.63, 1.68	0.68, 1.75
Other Store		1.13	1.14	1.12
		0.79, 1.66	0.79, 1.68	0.77, 1.64
Near School		1.16	1.16	1.12
		0.99, 1.36	0.99, 1.37	0.96, 1.33
Near TRO		**1.23**	**1.24**	**1.21**
		1.08, 1.40	1.08, 1.43	1.05, 1.39
SNAP		0.88	0.89	0.94
		0.75, 1.02	0.75, 1.04	0.79, 1.10
WIC		1.18	1.19	1.17
		0.96, 1.47	0.96, 1.51	0.94, 1.49
TRO Density		0.99	0.99	1.01
		0.94, 1.04	0.92, 1.06	0.95, 1.08
Smoking Rate		1.01	1.02	1.02
		0.95, 1.08	0.95, 1.09	0.93, 1.13
DIC	6841.6	6816.6	6770.4	6707.8
pD	3.9	16.0	198.0	137.7

Convenience store is the reference for store types. Significant effects are in bold.

Regarding inference on the factors in the neighborhood deprivation index, in the crude model index the weights were estimated to be 0.17 for percent Black population, 0.30 for percent of families in poverty, 0.20 for the Gini index of income inequality, 0.12 for percent without a bachelor’s degree, and 0.21 for inverse percent of Hispanic population. Therefore, indicators of poverty and income inequality received a majority of the weight in the index, followed by ethnicity, race, and education. Percent of families in poverty was also the most important component of the index in the adjusted and independent random effects models. Adding spatial random effects changed the importance rankings of the components according to the weights. In this model, inverse Hispanic population received the most weight (0.29) and the others ranged in weight from 0.17–0.18.

There were consistent findings about store and other area characteristics across the models. Regarding store type, the store types of pharmacy and grocery had significantly lower likelihood of a sales violation compared with convenience stores. Mass merchandisers were also associated with a lowered risk of a sales violation that was marginally significant. Tobacco shops, vape shops, and other stores all had increased likelihood of a sales violation compared with convenience stores. There was a significant positive association (OR = 1.21) between being located near another TRO and having a sales violation. Being located near a school was associated with an increased likelihood of a sales violation (OR = 1.12) but it was not significant. Accepting WIC was also associated with an increased but not significant likelihood of a sales violation (OR = 1.17). There was little evidence of an association between sales violations and scaled county smoking rate (OR = 1.02) and scaled TRO density at the census tract level (OR = 1.01).

**Fig 3:**
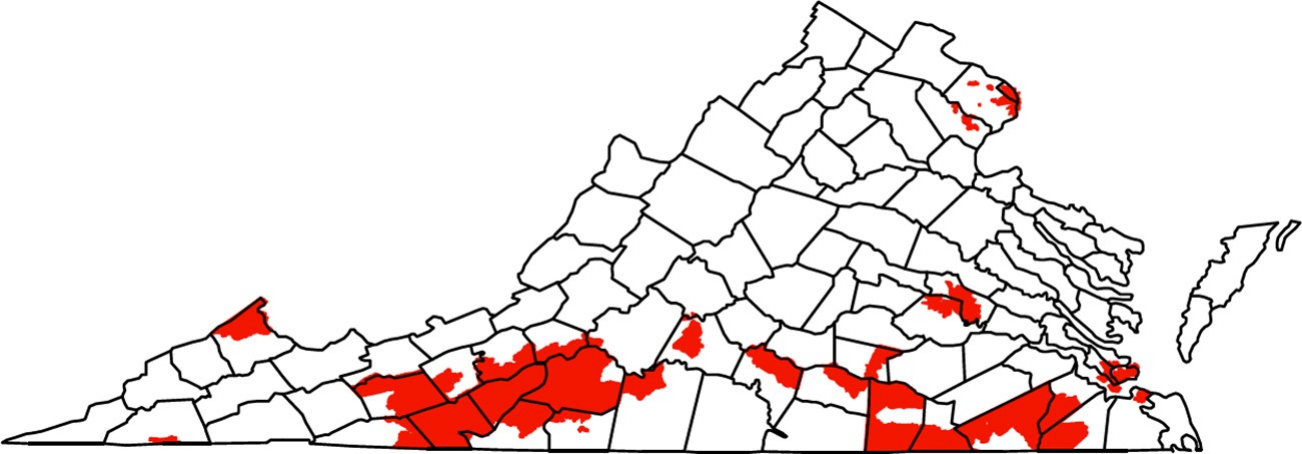
Census tracts most associated with increased likelihood of a TRO sales violation according to exceedance probabilities from the best fitting model with county boundaries included for reference.

According to the DIC, the spatial random effects were beneficial for explaining the likelihood of a sales violation. Using exceedance probabilities, we identified the census tracts most associated with increased likelihood of a sales violation (Fig 3). There is a large spatial cluster of these tracts in southwestern Virginia that covers many counties including Wythe, Pulaski, Floyd, Carroll, Patrick, Franklin, Roanoke, Pittsylvania, and Henry Counties. There is a small cluster in Buchanan County along the border with West Virginia. In addition, there are clusters in southeastern Virginia including Suffolk, Southampton, Greensville, and Brunswick Counties and the eastern shore area of Hampton and Newport News. There are also clusters around the cities of Richmond and Arlington.

## Discussion

In this study, we leveraged data on tobacco sales violations and linked these data with other publicly available area-level data to better understand trends in the timing and location of tobacco sales violations to minors and what explains the variation in violations. We identified a number of factors associated with a higher likelihood of tobacco sales violations, including store-type (primarily convenience stores with or without gas), being located near another retailer, and neighborhood deprivation. Our finding that sales violations vary considerably by store type is in agreement with the one other similar study that found that gas stations were most associated with increased illicit sales to minors [9]. Our results also showed significant spatial variation in the likelihood of retail sales violations, with statistically higher likelihood of sales violations occurring in specific areas of the state (primarily the southwest and southeast). Together, the results highlight the need to better understand where and why TRO sales violations are occurring, in order to improve efforts aimed at monitoring, educating, and remediating TRO sales violations.

The finding of significantly increased likelihood for sales violations for TROs located near other retailers is a new finding. Reasons for this are unknown, but may potentially have to do with retailers competing for tobacco sales, suggesting that potentially reducing tobacco density in certain markets may have the added benefit of reducing youth sales violations. We also found a positive and marginally significant association between TROs accepting WIC and sales violations. A previous study of the presence of tobacco marketing in retailers found significant positive associations between displaying interior tobacco price promotions and SNAP-authorized retailers (OR 2.3, 95% CI [1.7, 3.1]) and WIC-authorized retailers (OR 1.7, 95% CI [1.1, 2.4]) compared with non-WIC/SNAP retailers [29]. While we did not study tobacco advertising, retailers with interior price promotions may be suggestive of permissive sales environments leading to violations.

Regarding area-level variables, we found a significant positive association between neighborhood deprivation and sales violations in all models except when including spatial random effects. The index was composed of the Gini index of income inequality, percent Black population, percent without a bachelor’s degree, percent of families in poverty, and inverse Hispanic population. Indicators of poverty and income inequality received a majority of the weight in the index in crude and adjusted models, while the percent non-Hispanic received the most weight in the index when including spatial random effects. Previous studies have found positive associations between sales violations to minors and the proportion of the population living in poverty [13, 14, 16] and the proportion of population that is African American [13, 14, 17]. However, in a study of illicit sales to minors in Washington, DC, the area variables of the proportion of African American population in the block group and the proximity to public housing developments within 1 mile were not significantly associated with illicit sales to minors [9]. The only significant association related to area-level variables found in that study was an interaction between block groups with more than 56% African American population and distance to high school, where there was an inverse association between distance to school and likelihood of a sales violation. In our study, we did find an increased likelihood (OR = 1.12) of a sales violation for TROs within 1,000 feet of a school, but it was not statistically significant. Our finding of the importance of income inequality as a factor of neighborhood deprivation is a new finding in the literature for tobacco sales violations.

In addition to the findings related to area-level variables, we found significant spatial patterning in the likelihood of violations at the census tract level, with a particularly large spatial cluster of high risk in southwest Virginia. This area of the state, called the piedmont area, is where tobacco farming is most common, and as we have shown previously, is an area where there is a high density of tobacco retail outlets [20]. However, the density of TROs visited for compliance checks in our study period was not associated with likelihood of a sales violation.

Our study has a number of strengths, including a large and diverse geographic area, inclusion of data at multiple scales (e.g., store, census tract, county), and an assessment of geographic variability in sales violations. This is the first study to our knowledge to use Bayesian spatial modeling to evaluate neighborhood deprivation related to TRO sales violations and uncover geographic clusters of tobacco policy violations. This type of spatial risk modeling could inform precision public health strategies aimed at reducing youth sales violations. In addition, the use of sales violations at the individual store-level helps reduce ecological bias inherent in previously published literature on this topic and allows for a more comprehensive assessment of risk factors for sales violations including several store-level characteristics. Another strength is using all available compliance check data (i.e., considering multiple visits per TRO) over an extended time period and specifically modeling sales to minors as the violation type. A potential limitation is that inspection and compliance check outcomes depend on a number of factors that cannot be accounted for with available data, such as characteristics of the minor and retail clerk, circumstances surrounding the transaction, and frequency of compliance checks [30]. Additionally, we identified an increasing time trend in violation rate. Two factors may be contributing to this trend; namely, better monitoring and record keeping with the implementation of ground truthing Virginia’s tobacco retail outlets beginning in 2015 and a loss of FDA funding to support compliance checks in 2019. Further research is needed to disentangle the individual and joint effects of these factors on violation rate trends. Another limitation is that we used publicly available estimates of county smoking rates and do not have estimates of the variability of the rates.

Despite these limitations, the findings highlight a number of practical implications. We found that convenience stores have significantly higher violation rates than other store types (e.g., pharmacies and grocery stores) and make up the majority of the sales violations. Thus, inspections of convenience stores should be a higher priority for inspections and particularly those that are located near another tobacco retailer. In addition, those that accept WIC program benefits should also have higher priority for inspections because this factor was marginally related to greater violations. Further, we found uneven risk of a violations across the state, with significantly elevated clusters of sales violations in the southwest and southeast. Additional research across different geographies is needed to confirm the generalizability of our findings and extend them to identify spatial clustering of TRO sales violations and factors that may help explain these spatial patterns.

## Conclusions

Although federally mandated enforcement efforts by states to prevent the sale of tobacco to minors has been found to have made an important contribution to reducing smoking among youth, our study suggests the need for a more targeted approach to compliance checks to reduce TRO sales to minor violations. This is especially true in states, like Virginia and 12 other states, where no license is required to sell tobacco. Strong local tobacco retail licensing ordinances that provide adequate resources to fund regular compliance checks and enforcement have been found to reduce the use of cigarettes and other tobacco products among youth and young adults. Implementing these changes could help to change social norms related to tobacco use. If a targeted approach is taken, better tobacco retail licensing policies may also have the potential to reduce geographic disparities in youth tobacco use [31]. In the absence of such policies that are regularly enforced, targeted and continued inspections and interventions among stores and areas of higher risk should ultimately reduce access to tobacco products for minors and reduce this contributing factor to tobacco-related geographic disparities.
